# Increased mercury emissions from modern dental amalgams

**DOI:** 10.1007/s10534-017-0004-3

**Published:** 2017-02-20

**Authors:** Ulf G. Bengtsson, Lars D. Hylander

**Affiliations:** 1 Department of IEI, Linköping University, S-581 83 Linköping, Sweden; 2 Department of Energy and Technology, Swedish University of Agricultural Sciences, S-750 07 Uppsala, Sweden

**Keywords:** Mercury, Non-gamma-two, Non-ɣ2, Copper amalgam

## Abstract

All types of dental amalgams contain mercury, which partly is emitted as mercury vapor. All types of dental amalgams corrode after being placed in the oral cavity. Modern high copper amalgams exhibit two new traits of increased instability. Firstly, when subjected to wear/polishing, droplets rich in mercury are formed on the surface, showing that mercury is not being strongly bonded to the base or alloy metals. Secondly, high copper amalgams emit substantially larger amounts of mercury vapor than the low copper amalgams used before the 1970s. High copper amalgams has been developed with focus on mechanical strength and corrosion resistance, but has been sub-optimized in other aspects, resulting in increased instability and higher emission of mercury vapor. This has not been presented to policy makers and scientists. Both low and high copper amalgams undergo a transformation process for several years after placement, resulting in a substantial reduction in mercury content, but there exist no limit for maximum allowed emission of mercury from dental amalgams. These modern high copper amalgams are nowadays totally dominating the European, US and other markets, resulting in significant emissions of mercury, not considered when judging their suitability for dental restoration.

## Introduction

The vast majority of mercury containing fillings consists of two principal ingredients; liquid mercury and a metal powder referred to as the alloy. The mixing ratio is approx. 50 wt% of each with small variations, although alloys with high content of spherical alloy particles requires somewhat less mercury (Anusavice et al. 2012). This mixing is referred to as trituration by dental science.

The term alloy, when used in physics, refers to one or more elements, at least one being a metal, which are dissolved into each other. When used by dental science, alloy refers to a mixture of solid metal particles, not including mercury apart from very small amounts sometimes added (pre-amalgamation). When the bulk of mercury is added to the alloy powder, reactions take place and the resulting compound is called dental amalgam. Amalgams are mixtures of mercury and one or more other metals, which may be dissolved into the mercury or being metal particles just glued together by mercury (Hylander and Plath 2006). Silver, being the main component of the presently dominating alloy, has resulted in the name silver fillings of these restorations. Considering that mercury, not silver, is the dominating metal in the final filling, they should rather be termed mercury fillings.

The alloy/mercury mixing ratio is set by the manufacturer at a ratio, where the mercury has been claimed to be firmly bound to the alloy in the dental amalgam. Although this assumption has been proved to be erroneous (Homme et al. 2014), there is no consensus on acceptable emissions from dental fillings and there is no awareness of differences in mercury losses from conventional amalgams and non-ɣ2-amalgams, respectively. In addition, a limited number of dentists prefer a softer mix, using an increased amount of mercury. This is known in dental science as the “wet technique” (Möller 1978; Bergdahl 1973). The excess mercury will be removed in the oral cavity when the mix is squeezed/packed into the prepared tooth cavity. This squeezing out/packing is referred to as condensation by dental science but has nothing to do with the term as used in physics. The wet technique requires the use of bulk mercury and alloy. As a consequence of the ban on the use of bulk mercury in dentistry agreed upon in the Minamata Convention, this technique will be prohibited in the future. However, many manufacturers still provide bulk alloy and mercury. One manufacturer gives two alternative mixing ratios, 1:1 and 1:1.2, the latter suitable for dentists preferring the wet technique (Nordiska Dental 2017).

Study of the microstructure of the amalgam filling reveals that it is not homogenous, but it consists of a number of different phases; ɣ1, ɣ2, ɛ and more (Anusavice et al. 2012). Depending on the copper content, the fillings are termed either low copper amalgams or high copper amalgams. These expressions refer to the now withdrawn standards ISO 1559 Ed.1 and Ed.2, which stipulated 6% Cu max. and 30% Cu max., respectively. When increasing the copper content, the ɣ2-phase slowly disappears and at around 12%, it has almost disappeared. Amalgams with a copper content resulting in no ɣ2-phase are called non-ɣ2 amalgams (non-gamma-two).

The ɣ1-phase, present in both low and high copper amalgams, is transformed to the β1-phase with considerably less mercury. This phase transformation goes on for years constantly generating free mercury (Schmalz and Arenholt-Bindslev 2009; Mahler et al. 1973).

Standards for the composition below refers to the alloy ingredients, not the final filling material.

## Methods used and types of amalgams

This study is based on observations of droplets rich in mercury found on dental fillings, challenging the dominating assumption that mercury in amalgam is firmly bonded to the alloy. The observations were photographed with a light microscope (×252 magnifying), analyzed with a scanning electron microscope (SEM) and a literature review was performed to explain the phenomena and possible implications of these observations at the surface of dental amalgam fillings. The study includes two groups of dental amalgam: conventional amalgams and non-ɣ2-amalgams. Copper amalgam is included in the background description to clarify its specific properties.

### Conventional amalgams

Due to the fact that the alloy of conventional amalgams contains max. 6% copper, they are also known as low copper amalgams. These are rich in the ɣ2-phase, known for its poor corrosion resistance (Anusavice et al. 2012).

ISO 1559, 1st ed. 1978 (now withdrawn), stated:

- Silver: 65% min.
- Tin: 29% max.
- Copper: 6% max.
- Mercury: 3% max.
- Zinc: 2% max.

### Non-ɣ2-amalgams

The first one of these non-ɣ2-amalgams was patented by a Canadian inventor (Youdelis 1967). It later became known as Dispersalloy and its alloy partly contains particles with a spherical form. This spherical alloy for dental applications, used in many of today’s mercury fillings, was invented by the American Dental Association (Marjenhoff and George 1992). Grantees of the US Public Health Service was not allowed to protect their inventions before 1980, so the ADA never had the opportunity to patent it.

These new amalgams were initially not in accordance with the standard above, so ISO 1559 Ed. 2, 1986 (now withdrawn), was released updating the composition requirements to include alloys with high copper contents that already had been on the market for more than 10 years:

- Silver: 40% min.
- Tin: 32% max.
- Copper: 30% max.
- Mercury: 3% max.
- Zinc: 2% max.

The present standard is ISO 24234 Ed.2, 2015, and includes other compositions, which have been on the market in violation of ISO 1559 Ed.2:

- Silver: 40% min.
- Tin: 32% max.
- Copper: 30% max.
- Indium: 5% max.
- Palladium: 1% max.
- Platinum: 1% max.
- Zinc: 2% max.
- Mercury: 3% max.

The mercury in the alloy standards above is there to allow for pre-amalgamation to aid the final mixing, the trituration, with mercury.

ISO standards do not regulate the market for mercury fillings but products already on the market drive the development of these standards.

### Copper amalgam

One outdated member of the family of mercury containing filling materials is the copper amalgam. It must not be mistaken for the low or high copper versions mentioned above.

**Fig. 1:**
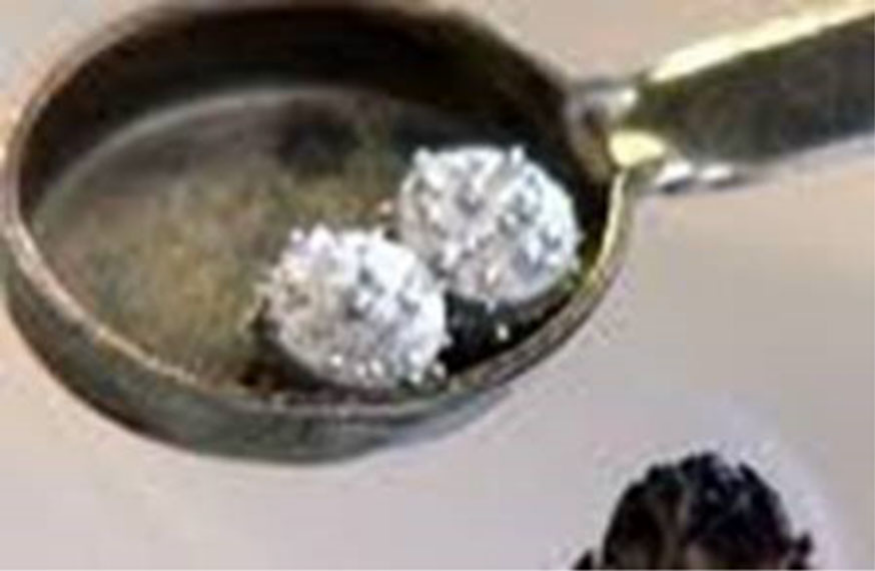
Two tablets of copper amalgam in a spoon heated over an open flame ready to be crushed. With courtesy of the Norwegian TV Company NRK

Copper amalgam is provided as small round or square tablets consisting of approx. 70% mercury and approx. 30% copper. Sometimes it is spiked with approx. 1% of cadmium (Örstavik 1985). Cadmium amalgam with cadmium and tin has been in use. It was discontinued when found that cadmium was one of the worst metals that could be used in a dental alloy and therefore already in 1849 recommended to not use (Hodgen 1924). When restoring a dental cavity with copper amalgam, small pieces of amalgam are placed in a spoon and heated over an open flame until droplets of mercury are visible on the surface of the metal, see Fig. 1.

The tablets are then crushed and triturated with pestle and mortar and allowed to cool and is then inserted into the prepared cavity. In the Nordic countries, it was predominantly used in children with extensive caries, but was sometimes also used in adults. The latest documented use in Sweden is from 1981 and in Norway it was used as late as 1994 (Kromberg and Röynesdal 1994). It was sold in Europe as late as 2001 (Produits Dentaires SA 2001).

Copper amalgam is known for its high corrosion rate, giving it increased antibacterial effects (Örstavik 1985). In a document from the Nordic Institute of Dental Materials (NIOM), the head of the institute calculates that a child with copper amalgams in all molars (10 g) could be exposed to 2.3 g of mercury and 1.0 g of copper annually in a worst case scenario (Mjör 1981).

Copper amalgam is still sold in India and the provider is also an exporter (Pyrax Polymars 2017). Even though its use may be limited, it is still regarded as a viable alternative by the Indian Dental Academy, a national leader in continuing dental education (Indian Dental Academy 2017). It is not mentioned in the Minamata Convention despite the fact that the use of copper amalgam is one of the few activities apart from Artisanal and Small-Scale Gold Mining (ASGM), where mercury is deliberately heated with extensive emission of mercury as a consequence.

The Indian company confirms that it sells copper amalgam with approx. 70% mercury in the form of tablets to be heated. In the package insert, it is however stated that the tablets consist of equal amounts of mercury and copper. If the latter is true, this is a new dental alloy not previously accounted for in the scientific literature (Pyrax Polymars 2017).

## Instability phenomena

### Droplets on the surface of non-ɣ2-amalgams

**Fig. 2:**
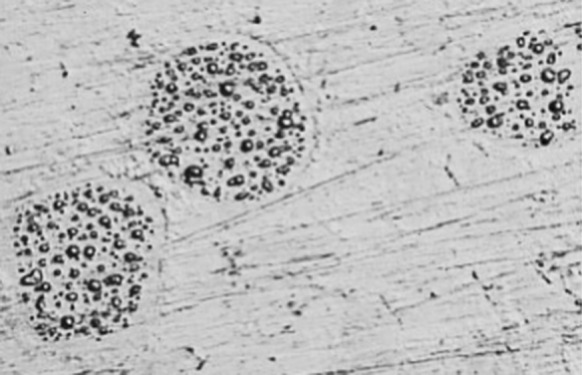
Droplets of mercury on the surface of modern, high copper non-ɣ2-amalgam, photographed with a light microscope (×252 magnifying). Photo: Ulf Bengtsson

**Fig. 3:**
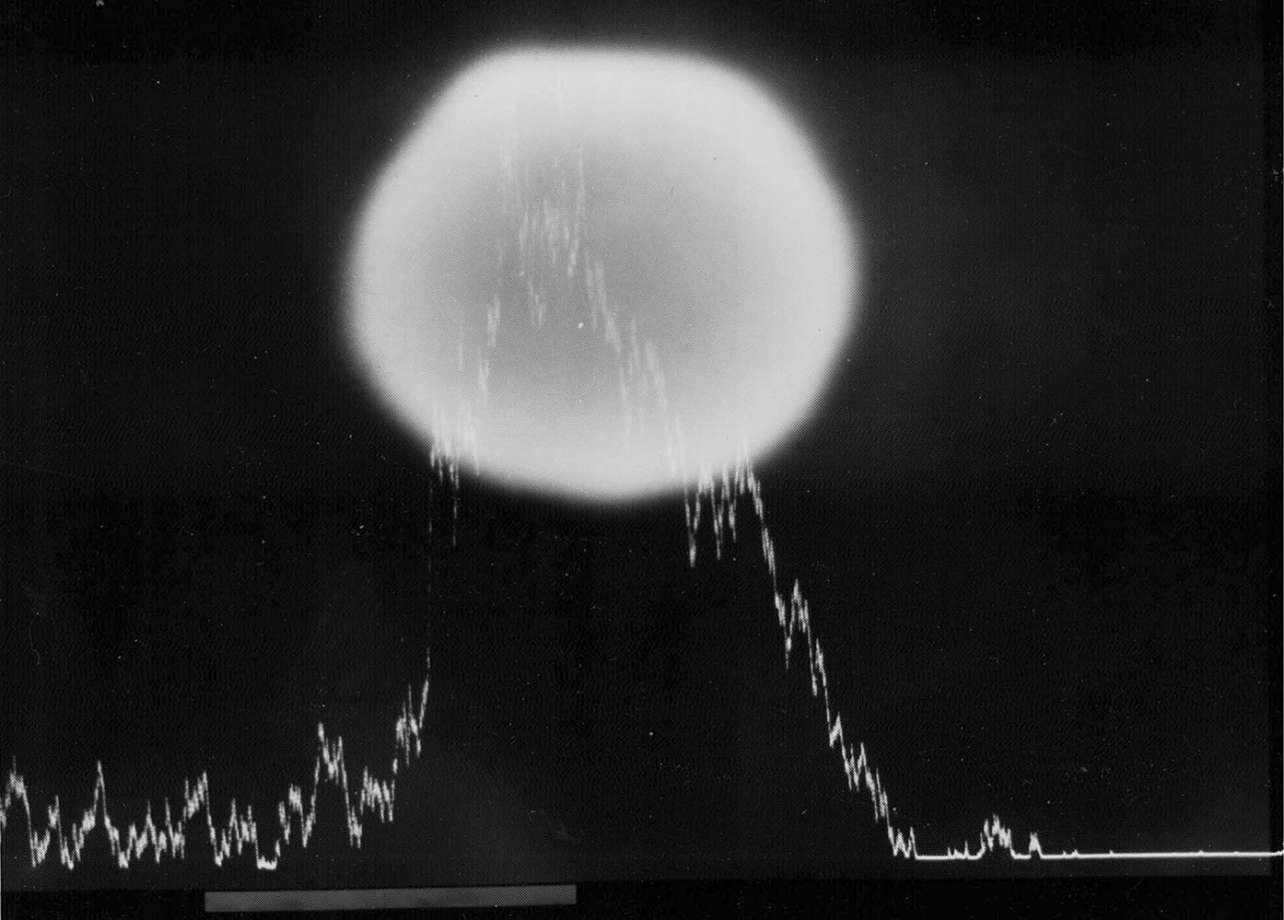
A sphere of mercury on the surface of modern, high copper non-ɣ2-amalgam, documented with a scanning electron microscope (SEM). Note the strong signal from mercury as the electron beam passes the sphere. Photo: Ulf Bengtsson

Polishing the surface of many high copper amalgams stimulates the formation of droplets rich in mercury, see Figs. 2 and 3. This formation happens even if the polishing takes place under cold water to avoid any rise in temperature and continues a number of hours after the polishing has stopped.

This phenomenon was first described by Rehberg and Scharschmidt in 1976 and has since been verified by a number of researchers (Rupp et al. 1979; Schneider and Sarkar 1982; Sarkar et al. 1991). Publication has mainly been done in the form of scientific meetings abstracts but to our knowledge no dental scientific journal paper has ever been devoted to this most striking phenomenon alone. Some abstracts are not even possible to get from the dental organization, who initially held the meetings. However, there has obviously been internal discussions taking place and some regard this as a polishing artefact. Observations of droplets have however been made on clinical fillings contradicting this notion (Fredin 1994).

One of the very few pictures of these droplets in the dental scientific literature can be seen in one of the big standard encyclopedias of dental materials accompanied by the text: “The small, very light, drop-shaped areas on particle phase are high in mercury owing to the freshly polished specimen (×1000).” (Anusavice et al. 2012). No further discussion of the phenomenon is offered. Another picture of droplets without comment is presented by Herö et al. (1983).

A few papers, published outside of the dental community, have however dealt with this phenomenon. Both the formation of droplets and documentation of them is presented by a corrosion expert, outside of the dental community (Pleva 1994).

In another study, the investigator has indeed seen small “globules” on all surfaces of fillings from extracted teeth, indicating that this is not just an in vitro phenomenon but indeed occurs in clinical situations. Unfortunately the type of amalgam was not accounted for (Fredin 1994).

In 1985, one of the authors (UB) contacted the National Board of Health and Welfare in Sweden about findings of droplets on the surface of modern amalgams. The Swedish Institute for Metals Research was given the task of stripping these small droplets from the surface to determine their content of mercury. Through an extraction replica technique, five droplets were lifted from the surface and measurements ranged from 44.1 to 85.4% mercury (Lehtinen 1985).

These findings gave rise to the suspicion that the formation of these droplets was accompanied by an increased emission of mercury vapor. A final examination project was initiated at Linköping University to study mercury vapor emission in amalgams, previously found to produce droplets, with low copper amalgams used as controls. It was concluded that, indeed, non-ɣ2 amalgams exhibit an increased emission of mercury vapor (Toomväli 1988).

One would expect that droplets rich in mercury found on high copper fillings should have been published and discussed in journals commonly read by dental personnel, especially in an issue involving safety. As far as we can find, this has not happened.

This is one of two phenomena of instability, introduced with the new non-ɣ2-amalgams. The other is described below.

### Increased emission of mercury vapor in non-ɣ2-amalgams

**Fig. 4:**
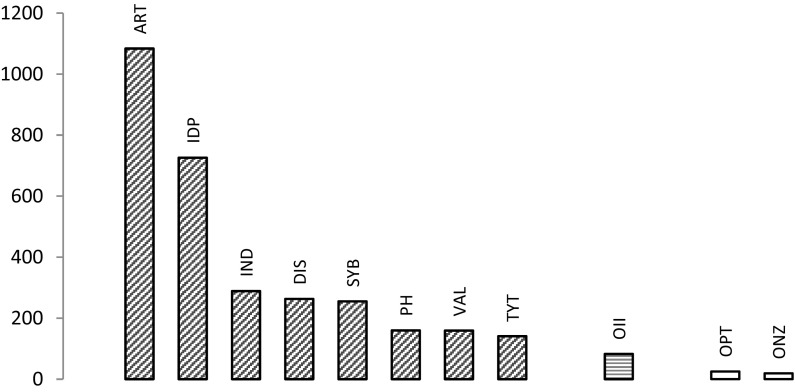
Mercury vapour loss (ng) between 0.5 and 30 min after abrasion. Left group (*red cross-hatched bars*): non-ɣ2-amalgams; third bar from right (*blue hatched*): reduced ɣ2-amalgam; right group (*two white bars*): old, conventional ɣ2–containing amalgams Diagram based on findings in Mahler et al. (1994). (Color figure online)

In 1994, it was shown that the amount of tin in the ɣ1-phase is related to the emission of mercury vapor (Mahler et al. 1994). Based on this paper, it is possible to identify the brands tested: conventional amalgams, amalgams with reduced amount of ɣ2- and non-ɣ2-amalgams. The result is clear; non-ɣ2-amalgams emit substantially more mercury vapor than the old, conventional ones used before the 1970s, see Fig. 4. Using the highest emitter of the low copper amalgams as a baseline, the high copper amalgams emits 3–43 times as much mercury vapor depending on brand. One of the most wide spread amalgams, DIS, emits ten times the amount of mercury vapor as compared with the highest emitter of the conventional amalgams, OPT, under the experimental conditions used.

Also Ferracane (1995) compared losses of mercury as related to the amount of ɣ1-phase. He confirmed the pattern of differences in mercury vaporization from amalgams of different composition. Using the highest emitter of the low copper amalgams as a baseline, the high copper amalgams emitted 3–62 times as much mercury vapor depending on brand and the high copper amalgams had by far the highest emission of mercury vapor (Ferracane 1995).

In an investigation measuring differences in mercury vapor emission in corroded and uncorroded samples, only one non-ɣ2-amalgam and one low copper amalgam was used. The pattern is once again confirmed with the non-ɣ2-amalgam emitting substantially more mercury vapor than the conventional one (Boyer 1988). Corroded samples emitted more mercury vapor than not corroded ones (Boyer 1988). In another investigation, using the same brands of amalgam as Mahler et al. (1994), the specimens were abraded, immersed in artificial saliva and mercury was then measured in the solution after 2h (Marek 1997). Also in this investigation, the mercury loss decreased with increasing tin content in the ɣ1-phase. In a second part of the test, when the specimens were treated differently in order to generate an oxide layer before testing, there was no relation between mercury loss and tin content. 

In the four investigations above, the main researchers in dental amalgam are all reaching similar results. When the reducing oxide layer is removed, the emission of mercury is inversely related to the amount of tin in the gamma-1 phase. This oxide layer is very fragile, so touching the surface with a piece of cotton wool will result in higher levels of mercury vapor.

Unfortunately, we cannot find any openly published information/discussion on increased emission of mercury vapor from modern amalgams in any journal commonly read by dental personnel. On the contrary, several big national and international dental organizations have stated that mercury fillings are stable.

Thereby, this is the second phenomenon of instability, introduced with the new non-ɣ2-amalgams, which needs to be considered when evaluating exposure and losses of mercury from dental amalgam. Increased emission of mercury vapor may be provoked by a slight touch of the filling surface as by chewing or polishing or by a slight increase of temperature such as consuming hot beverages or hot food.

## Conclusion

The non-ɣ2-amalgams are marketed as superior in strength and corrosion resistance. When trying to meet these goals for development, a strong sub-optimization has occurred. In experimental set ups, these amalgams, being introduced in the 1970s, emit about ten times more mercury vapor than the ones previously used. Ordinary dental personnel, politicians and other decision makers has not been informed about the instability of modern non-ɣ2-amalgams.
